# Trends of Colorectal Cancer Incidence in Kazakhstan

**DOI:** 10.31557/APJCP.2021.22.10.3405

**Published:** 2021-10

**Authors:** Dana Mauyenova, Yerkezhan Zhadykova, Arman Khozhayev, Dulat Turebayev, Dariyana Kulmirzayeva, Saltanat Urazova, Gaukhar Nurtazinova, Yerlan Kuandykov, Akmaral Amanshayeva, Sauirbay Sakhanov, Zhanar Bukeyeva, Vladimir Openko, Serikbay Orazbayev, Saken Kozhakhmetov, Zarina Bilyalova, Gulnur Igissinova, Alireza Mosavi Jarrahi, Nurbek Igissinov

**Affiliations:** 1 *Astana Medical University, Nur-Sultan, Kazakhstan. *; 2 *Central Asian Cancer Institute, Nur-Sultan, Kazakhstan. *; 3 *Department of Oncology, Asfendiyarov Kazakh National Medical University, Almaty, Kazakhstan.*; 4 *National Center for Neurosurgery, Nur-Sultan, Kazakhstan. *; 5 *Khoja Akhmet Yassawi International Kazakh-Turkish University, Turkistan, Kazakhstan. *; 6 *Cancer Research Center, Shahid Beheshti University of Medical Sciences, Tehran, Iran. *; 7 *Eurasian Institute for Cancer Research, Bishkek, Kyrgyzstan. *

**Keywords:** Colorectal cancer, incidence, trends- component analysis, Kazakhstan

## Abstract

**Background and objective:**

Colorectal cancer (CRC) remains one of the most widespread human malignancies. The aim of this study was to study trends of the incidence of CRC in Kazakhstan.

**Materials and Method:**

This retrospective study was done using descriptive and analytical methods of oncoepidemiology.

**Results:**

During the study period from 2009 to 2018, 28,950 new cases of CRC were recorded, 13,779 (47.6%) cases were allocated to men and 15,171 (52.4%) to women. It was found that the incidence rate of CRC increased from 14.79 (2009) to 17.72 in 2018 and the overall growth was 2.93 cases per 100,000. This increase was due to the age structure – ∑Δ_A_=+1.42, the risk of acquiring illness – ∑Δ_R_=+1.31, and their combined effect – ∑Δ_RA_=+0.20. The component analysis results revealed that the increase in the number of patients with CRC was mainly due to the growth of the population (ΔP=+37.7%), changes in age structure (Δ_A_=+26.6%), and changes associated with the risk of acquiring illness (Δ_R_=+24.6%). The number of patients (both sexes) was increasing in many regions largely due to the influence of the age structure of the population. In addition, it was found that growth in the number of patients in most regions, both men and women, was associated primarily with the risk of acquiring illness.

**Conclusion:**

The findings of the current study showed increasing trends in the incidence of CRC in all regions of the country. These changes were mainly influenced by demographic factors (population size and age structure), risk of acquiring the disease, and their combined effect.

## Introduction

Colorectal cancer (CRC) remains one of the most widespread human malignancies. According to the International Agency for Research on Cancer, about 1.93 million new cases of CRC were registered in 2020 worldwide, and the age-standardized incidence rate (ASR) of CRC is 19.5 per 100,000. About 50.3% of all new CRC cases occur in Asia and its incidence is 17.6 per 100,000 in this region (Ferlay et al., 2020A). According to Global Cancer Observation, the number of new CRC cases in the world will increase by 63% in 2040 (about 3.15M) (Ferlay et al., 2020B).

There is a wide geographic variation in CRC incidence (Ferlay et al.,2020) due to dietary characteristics (Baena and Salinas, 2015; Yang and Yu, 2018), bad habits (Fliss-Isakov et al., 2018), environmental factors, and genetically driven susceptibility (Johnson et al., 2013; Gu et al.,2018; Keum and Giovannucci, 2019; Mattiuzzi et al., 2019).

In the recent decades, dietary and food consumption patterns of the population have changed (Chun et al., 2010; Kearney, 2010). For instance, there have been many changes in water consumption (Muckelbauer et al., 2016), food processing and storage technologies (Orlien and Bolumar, 2019), and the use of preservatives, stabilizers, aroma enhancers, synthetic dyes, and other food additives (Eskola et al., 2020).

In agriculture sector, different technologies are used to accelerate production of breeding material. In addition, hormonal drugs and antibiotics are used (Liu et al., 2017; Oliveira et al., 2020), most likely leading to a change in the normal human microbiota and malignant transformation of cells (Murphy et al., 2019).

CRC develops as a result of degeneration of adenomatous polyps (Lucas et al., 2017). Two of the main risk factors associated with CRS are hereditary (Mármol et al., 2017) and familial and factors (Valle, 2017; Boland et al., 2018). Other known predisposing factors include inflammatory bowel disease (ulcerative colitis, Crohn’s disease) and the duration of the disease (Nadeem et al., 2020).

The overall incidence of CRC begins to increase approximately 8-10 years after the onset of inflammatory bowel disease and increases to 15-20% after 30 years (Eaden et al., 2001). The main risk factors are the duration of the disease, the prevalence of the lesion, young age, and the presence of complications (Dyson and Rutter, 2012). Age is a significant risk factor for the development of CRC. CRC is rare until the age of 40, but the incidence of CRC increases in each subsequent decade and peaks at 60-75 years (Siegel et al., 2017).

 The high frequency of CRC is attributed to low fiber, high animal protein, fat, and refined carbohydrate food content (Baena and Salinas, 2015). Obesity increases the risk of CRC by about 1.5 times or more in men (Dong et al., 2017). Alcohol consumption, smoking, and inherited colon diseases also increase the sporadic incidence of colon polyposis and CRC (Amitay et al., 2020).

Screening tests significantly reduce CRC incidence by detecting precancerous bowel disease or the cancer at early stage, allowing for timely medical treatment (Rex et al., 2017; Lauby-Secretan et al., 2018; Ladabaum et al., 2020). WHO recommends preventive examinations, namely doing fecal occult blood test every year from the age of 50. In case of a positive test result, a more detailed examination of the colon, such as colonoscopy, should be carried out (Winawer et al., 1995). Since 2011, screening for CRC has been carried out in Kazakhstan among the entire population aged 50 to 70 every two years using fecal occult blood test and colonoscopy procedure.

This is a continuation of several studies of the dynamics in malignant neoplasms incidence by the method of component analysis, we conducted studies in this direction earlier (Igissinov et al., 2012; Igissinov et al., 2013; Igissinov et al., 2015; Kuanyshkaliyeva et al., 2016; Igissinov et al., 2019), assessing the influence of demographic factors, risk factors and their combination at the same time, taking into account gender, ethnic groups in different localities. The aim of the study was to use principal components analysis to shed light on the dynamics of the incidence of CRC in Kazakhstan.

## Materials and Methods

*Patient recruitment*

The cancer registry of the population of Kazakhstan covers 14 regions and cities of Almaty and Astana (now the city of Nur-Sultan). New cases of CRC were extracted from case report forms presented by the Ministry of Healthcare of the Republic of Kazakhstan (form 7 and form 35) from 2009 to 2018 (International Classification of Diseases – 10, , 10th Revision code C18-21).

*Population denominators*

The population of Kazakhstan was 18.2 million in 2018. Population denominators for the calculation of incidence rates from 2009 to 2018 were provided by the National Bureau of Statistics of Kazakhstan. At the same time, data on the number of populations of the republic, taking into account the studied regions, are used, all data are presented on the official website (Bureau of National Statistics, 2021).

*Statistical analysis*

Age-specific incidence rates (ASIRs) were calculated for eighteen different age groups (0-4, 5-9, …, 80-84, and 85+) using the world standard population proposed by WHO (Ahmad et al., 2001) and according to the recommendations developed by the National Cancer Institute (NCI, 2013). The extensive, crude (CR) and ASIRs were determined based on the generally accepted methodology used in sanitary statistics Accordingly, the annual averages (M, P), mean error (m), Student criterion, 95% confidence interval (95% CI), and average annual upward/downward rates (T, %) were calculated (Merkov and Polyakov, 1974; Glanz, 1998; dos Santos Silva, 1999; Tango, 2010; Merabishvili, 2015). Trends were determined using the least squares method, and the average annual growth rates were calculated using the geometric mean.

The dynamics of indicators was investigated using component analysis according to methodological recommendations (Dvoyrin and Aksel, 1987; Chissov et al., 2007). The component method was used in this study to decompose the increase in the number of cases in the same population, but at different time periods. According to this method, 7 components of the increase in the number of cases were considered. The first 3 components were associated with changes in the population size, its age structure, and the combined influence of these factors. The true increase in the number of patients was due to the change only in the indicator of the risk of acquiring illness and was represented by the 4th component. The next 3 components were associated with the risk of acquiring illness, an increase in the population, a change in its age structure, and the influence of all three factors. Thus, the last 4 components were associated with an increase in the risk of developing the disease. By “risk of acquiring illness”, we meant the whole complex of reasons that could lead to an increase, decrease, or stabilization of the incidence.

Reviewing and processing of the received materials was carried out using the Microsoft 365 software package (Excel, Word, PowerPoint). In addition, online statistical calculators were used (https://medstatistic.ru/calculators/averagestudent.html), where Student criterion was calculated when comparing the average values.

*Ethics approval*

Since this study involved the analysis of publicly available administrative data and did not involve contacting individuals, obtaining permission from an ethics committee was not required.

## Results

During the study period, 28,950 new cases of CRC were registered in the country (13,779 (47.6%) in men and 15,171 (52.4%) in women). The highest proportion of CRC patients (both sexes) aged 60 to 69 years old (60-64 years old:15.9% and 65-69 years old: 15.5%) (Table 1).

Age-related indicators of the incidence of CRC (per 100,000) peaked in the age group of 75-79 years in both sexes (151.9±4.2) ( male :205.6±6.2 , female :124.7±4.2) (Table 1).

Trends of ASIR of CRC in the entire population tended to increase in almost all age groups, except for the age group of 40-44 years old (T=−0.1%), 50-54 years old (T=−0.7%), and 70-74 years old (T=−0.2%). Trends of ASIR in the male population decreased in the age group of 75-79 (T=−0.4%), and 85 , and older (T=−1.5%). In the female population, the age indicators decreased in the age group of 30-34 (T=−1.0%), 40-44 (T=−1.9%), 50-54 (T=−2.2%), 55-59 (T=−0.7%), and 70-74 (T=−0.6%). It should be noted that the value of the accuracy of the approximation of the listed decreases was not significant (Table 1).

Trends of age indicators generally affected the overall incidence rates, so the crude rate of CRC incidence in the total population of the country increased from 14.79 (2009) to 17.72 in 2018 (p=0.000), the total increase was 2.93 per 100,000 (Table 2) and depended on changes (per 100,000) in the age structure of the population (∑Δ_A_=+1.42), the risk of acquiring illness (∑Δ_R_=+1.31), and the combined influence of the risk of acquiring illness and the age structure (∑Δ_RA_=+0.20). At the same time, the average annual growth rate of the aligned indicator was T=+2.0%, and the approximation confidence value was close to 1 (R^2^=0.7327).

In the male population of the republic, the CRC also increased from 14.69 (2009) to 17.69 in 2018 per 100,000, indicating a statistically significant difference (p=0.000). The overall increase (+3.00 per 100,000) depended mainly on changes in the age structure of the population (∑Δ_A_=+1.72) and the risk of acquiring illness (∑Δ_R_=+1.24). However, their combined effect was not pronounced (∑Δ_RA_=+0.04) (Table 2). The average annual growth crude incidence rate was T=+2.6% and the approximation value was R^2^=0.7356 (Table 1).

In the female population of the country, the overall increase (+2.87) in crude incidence rates from 14.89 (2009) to 17.76 per 100,000 (2018) (p=0.000) depended on changes in the age structure of the population (∑Δ_A_=+1.20), the risk of acquiring illness (∑Δ_R_=+1.33), and the combined effect of the risk of acquiring illness and the age structure (∑Δ_RA_=+0.34) (T=+1.4; R^2^=0.5218) (Tables 1 and 2).

Furthermore, we used principal components analysis to shed light on the dynamics of the incidence of CRC in Kazakhstan (Tables 3 and 4). The results showed that the increase in the number of patients with CRC in the republic was associated with the influence of the following factors:

1. Growth of population number ΔP=+37.7% (male – ΔP=+37.8%; female – ΔP=+37.5%).

2. Changes in the age structure of the population Δ_A_=+26.6% (male – Δ_A_=+31.2%; female – Δ_A_=+23.1%).

3. Combined effect of changes in population number and its age structure Δ_PA_=+3.6% (male – Δ_PA_=+4.4%; female – Δ_PA_=+3.0%).

4. Change in the risk of acquiring illness Δ_R_=+24.6% (male – Δ_R_=+22.5%; female – Δ_R_=+25.7%).

5. Combined effect of changes in the risk of acquiring illness and population number Δ_RP_=+3.3% (male – Δ_RP_=+3.2%; female – Δ_RP_=+3.4%). 

6. Combined effect of changes in the risk of acquiring illness and age structure of the population Δ_RA_=+3.7% (male – Δ_RA_=+0.7%; female – Δ_RA_=+6.6%).

7. Combined effect of the changes in the risk of acquiring illness, population number, and its age structure Δ_RA_P=+0.5% (male – Δ_RA_P=+0.1%; female – Δ_RA_P=+0.9%).

The total increase in the absolute number of new cases of CRC (both sex) was equal to the sum of the components: n2−n1=322+227+31+210+29+32+4=854 or +36.1% (Table 4) in comparison with the primary number of patients (854÷2364×100=36.1%). Accordingly, the components of the increase as a percentage of the initial level was equal to: 



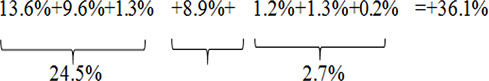



Thus, CRC (both sexes) was characterized by an increase in the number of cases because of the changes in the total population size and its structure (24.5% of the total increase of 36.1%). The real increase in the number of cases (risk of acquiring illness) was Δ_R_=+8.9%.

The increase in new cases of CRC in men and women is presented in Table 4, indicating no significant difference with respect to sex.

It should be noted that during the study period, the number of patients (both sexes) in the country increased by 36.1% (from 2,364 to 3,218), which was higher than the estimation obtained by the component analysis (– 2,945) (Table 3). The increase was due to demographic factors (Δ_P_+A+P_A_=+67.8%) and the combined influence of risk factors for acquiring illness with demographic components (Δ_R_+R_P_+R_A_+R_AP_=+32.2%). In men, the influence of demographic factors was slightly more pronounced (Δ_P_+A+P_A_=+73.5%), while the combined influence of risk factors and demographic factors was less pronounced (Δ_R_+R_P_+R_A_+R_AP_=+26.5%). Among women, the impact of demographic indicators led to an increase in patients (Δ_P_+A+P_A_=+63.5%), and the combined influence of risk factors and changes in demographic indicators also played a role (Δ_R_+R_P_+R_A_+R_AP_=+36.5%).

The dynamics of the incidence of CRC had regional characteristics. So that, in the Kostanay region, there was an overall increase (+9.42) in the incidence of CRC (per 100,000) in the entire population, from 23.02 in 2009 to 32.43 in 2018 (p=0.000) (Table 5), which primarily depended on the risk of acquiring illness (∑Δ_R_=+5.63 per 100,000) and secondly on changes in the age structure of the population (∑Δ_A_=+3.89 per 100,000). In contrast, the combined effect of the age structure and the risk of acquiring illness reduced this indicator (∑Δ_RA_=−0.11 per 100,000). At the same time, the average annual growth rate of the aligned indicator was Т=+3.0%, and the confidence value of the approximation was equaled to R^2^=0.5708. Having studies the role of various components, it was found (Table 5) that the incidence of new cases in this region was associated with demographic factors (Δ_P_+A+P_A_=+39.5%) and the complex influence of the risk of acquiring illness (Δ_R_=+62.4%) with the components of the population size, its age structure, and the influence of all the three above-mentioned factors (Δ_R_+R_P_+R_A_+R_AP_=+60.5%).

Following the analysis of the average annual growth rate of aligned indicators of CRC incidence in the entire population, it was detected that the most pronounced growth was in the Akmola (Т=+4.7%; R^2^=0.7630) and Atyrau regions (Т=+5.4%; R^2^=0.6244). This growth was statistically significant in 2018 in comparison with 2009, and the values of the accuracy of the approximation were pronounced (Table 5).

Regarding the influence of various components with respect to region for the entire population (Table 5), it was found that there was a pronounced decrease in the North Kazakhstan region (Δ_P_=−121.7%) due to changes in the population size, and the largest increase was allocated to Almaty city (Δ_P_=+98.6%). The role of the influence of age structure in the increase in the number of patients was positive in all regions, but it was mostly pronounced in the Pavlodar (Δ_A_=+281.6%) and North Kazakhstan (Δ_A_=+373.0%) regions. The combined effect of changes in the population size and its age structure showed a decline only in the North Kazakhstan region (Δ_PA_=−24.3%), while there was an increase in other regions, especially in Kyzylorda (Δ_PA_=+9.1%) and Mangystau (Δ_PA_=+10.3%) regions, as well as in Astana city (Δ_PA_=+9.9%). The decrease in the absolute number of patients with CRC due to the risk of acquiring illness was most pronounced in North Kazakhstan (Δ_R_=−180.4%) and Pavlodar (Δ_R_=−274.6%) regions, and the maximum increase was found in Atyrau (Δ_R_=+53.2%), Almaty (Δ_R_=+53.5%), and Kostanay (Δ_R_=+62.4%) regions. A pronounced increase in the combined impact of the risk of acquiring illness and the population size was found in Atyrau (Δ_RP_=+11.7%) and North Kazakhstan (Δ_RP_=+11.8%) regions. Changes in the risk of acquiring illness and the age structure led to a sharp decrease in the number of patients in the North Kazakhstan region (Δ_RA_=−169.4%), and the maximum rise was noted in Pavlodar region (Δ_RA_=+55.1%). In the North Kazakhstan region, the increase in the number of patients was highest compared to other regions due to the combined influence of the risk of acquiring illness, population size, and age structure (Δ_RA_P=+11.0%) .

In a nutshell, the component analysis revealed geographical variability in the dynamics of the number of patients and the incidence of CRC in Kazakhstan, which was associated with a difference in the influence of demographic factors (changes in population size, its age structure) and the risk of acquiring illness (i.e., a set of reasons that led to an increase, decrease, or stabilization of the rates).

**Table 1:** Number and Age-Specific Incidence rate of CRC in Kazakhstan, 2009-2018

Age	All				Male				Female			
	Number (%)	Incidence			Number (%)	Incidence			Number (%)	Incidence		
		per 100,000	Т, %	R^2^		per 100,000	Т, %	R^2^		per 100,000	Т, %	R^2^
<30	224 (0.8)	0.26±0.02	−1.6	0.0672	127 (0.9)	0.29±0.02	−1.6	0.0601	97 (0.6)	0.23±0.03	−1.5	0.0121
30-34	269 (0.9)	2.0±0.2	0.4	0.0026	136 (1.0)	2.1±0.2	1.8	0.0411	133 (0.9)	2.0±0.2	−1.0	0.0138
35-39	473 (1.6)	4.0±0.2	2.1	0.2455	233 (1.7)	4.0±0.2	0.2	0.0009	240 (1.6)	3.9±0.2	4.0	0.4537
40-44	819 (2.8)	7.4±0.3	−0.1	0.0008	418 (3.0)	7.8±0.3	1.6	0.1484	401 (2.6)	7.0±0.4	−1.9	0.093
45-49	1381 (4.8)	13.0±0.4	1.1	0.1374	674 (4.9)	13.3±0.6	1.6	0.1089	707 (4.7)	12.6±0.5	0.6	0.0222
50-54	2558 (8.8)	25.5±0.7	−0.7	0.0565	1236 (9.0)	26.4±0.7	1	0.1412	1322 (8.7)	24.7±1.1	−2.2	0.2749
55-59	3891 (13.4)	47.4±1.1	0.3	0.0129	1927 (14.0)	52.3±1.8	1.3	0.1287	1964 (12.9)	43.4±1.4	−0.7	0.0555
60-64	4614 (15.9)	77.0±1.6	0.3	0.0165	2273 (16.5)	89.7±3.1	0.4	0.0165	2341 (15.4)	67.7±1.2	0.1	0.0027
65-69	4488 (15.5)	113.1±6.1	3.7	0.4611	2189 (15.9)	138.1±7.8	4.7	0.6615	2299 (15.2)	96.7±5.5	2.8	0.2401
70-74	4240 (14.6)	128.2±2.4	−0.2	0.0091	1988 (14.4)	163.3±3.5	0.4	0.0287	2252 (14.8)	107.8±2.3	−0.6	0.0788
75-79	3746 (12.9)	151.9±4.2	0.3	0.0107	1696 (12.3)	205.6±6.2	−0.4	0.0201	2050 (13.5)	124.7±4.2	1	0.0883
80-84	1648 (5.7)	127.5±5.7	2.9	0.4099	644 (4.7)	169.5±10.2	3.5	0.3262	1004 (6.6)	110.0±5.1	2.2	0.2263
85+	599 (2.1)	81.1±3.7	0.1	0.0009	238 (1.7)	131.6±9.4	−1.5	0.0431	361 (2.4)	64.8±4.5	0.5	0.0046
CR	28950 (100.0)	16.9±0.4	2	0.7327	13779 (100.0)	16.7±0.5	2.6	0.7356	15171 (100.0)	17.2±0.3	1.4	0.5218
ASR		18.1±0.3	1	0.3182		21.9±0.5	1.4	0.4062		15.9±0.1	0.4	0.0724

T, average annual upward/downward rates; R^2^, the value of the approximation confidence; CR, crude rate; ASR, age-standardized rate

**Table 2:** Component Analysis of CRC Incidence Growth in Kazakhstan, 2009-2018

Age group (i)	Age structure		Growth of structural indicators *(S*_i2_*−S*_i1_*)* (3)−(2)	Incidence, per 100,000		Incidence growth, per 100,000			
						General *(P*_i2_*−P*_i1_*)* (6)−(5)	Including due to changes of		
	2009 *(S*_i1_*)*	2018 *(S*_i2_*)*		2009	2018		Δ_A_	Δ_R_	Δ_RA_
				*(P*_i1_ *)*	*(P*_i2_ *)*		(4)×(5)	(2)×(7)	(4)×(7)
1	2	3	4	5	6	7	8	9	10
Both sexes									
<30	0.5217	0.5011	−0,0206	0.29	0.23	−0,06	−0,006	−0,031	+0,001
30-34	0.0761	0.0837	0.0075	1.97	2.5	0.53	0.015	0.04	0.004
35-39	0.0711	0.0699	−0.0012	3.35	4.34	0.99	−0.004	0.07	−0.001
40-44	0.0669	0.0634	−0.0036	6.82	8.35	1.52	−0.024	0.102	−0.005
45-49	0.0689	0.0589	−0.0100	13.25	13.08	−0.17	−0.133	−0.011	0.002
50-54	0.0552	0.0559	0.0008	22.12	25.11	2.99	0.017	0.165	0.002
55-59	0.0432	0.0541	0.0109	46.51	45.84	−0.66	0.506	−0.029	−0.007
60-64	0.0256	0.0399	0.0143	75.78	75.45	−0.32	1.086	−0.008	−0.005
65-69	0.0252	0.0295	0.0043	79.89	111.19	31.3	0.344	0.789	0.135
70-74	0.0227	0.0144	−0.0083	127.66	122.18	−5.48	−1.061	−0.124	0.046
75-79	0.0117	0.0159	0.0042	130.22	137.95	7.73	0.542	0.091	0.032
80-84	0.0082	0.0086	0.0004	125.41	158.03	32.62	0.049	0.267	0.013
85+	0.0034	0.0048	0.0013	72.63	65.77	−6.86	0.096	−0.024	−0.009
Total	*∑S*_i1_ *=1.0*	*∑S*_i2_ *=1.0*		*P*_1_ *=14.79*	*P*_2_ *=17.72*	2.93	*∑Δ*_A_ *=+1.42*	*∑Δ*_R_ *=+1.31*	*∑Δ*_RA_ *=+0.20*
Male*									
Total	*∑S*_i1_ *=1.0*	*∑S*_i2_ *=1.0*		*P*_1_ *=14.69*	*P*_2_ *=17.69*	3	*∑Δ*_A_ *=+1.72*	*∑ΔR=+1.24*	*∑Δ*_RA_ *=+0.04*
Female*									
Total	*∑S*_i1_ *=1.0*	*∑S*_i2_ *=1.0*		*P*_1_ *=14.89*	*P*_2_ *=17.76*	2.87	*∑Δ*_A_ *=+1.20*	*∑Δ*_R_ *=+1.33*	*∑Δ*_RA_ *=+0.34*

Δ_A_, the age structure of the population; Δ_R_, risk of acquiring illness; Δ_RA_, risk of acquiring illness and age structure of the population; *The calculations were made in the same way as for the entire population.

**Table 3:** Component Analysis of the Dynamics of CRC Incidence in Kazakhstan from 2009 till 2018

Age group *(i)*	Number *(n*_ij_*)*		Population number *(N*_ij_*)*		Crude incidence *(P*_ij_^C^*)*		Standardized		END in 2018
	2009 *(j=1)*	2018 *(j=2)*	2009 *(j=1)*	2018* (j=2)*	2009 *(j=1)*	2018 *(j=2)*	2009 *(j=1)*	2018* (j=2)*	*(P*_ij_ *N*_i2_ *10* ^-5^ *) *(6)×(5)×10^-5^
1	2	3	4	5	6	7	8	9	10
Both sex									
<30	24	21	8338308	9099474	0.29	0.23		0.12	26.2
30-34	24	38	1216653	1519070	1.97	2.5		0.19	30
35-39	38	55	1135971	1268564	3.35	4.34		0.308	42.4
40-44	73	96	1069726	1150288	6.82	8.35		0.559	78.5
45-49	146	140	1101902	1070014	13.25	13.08		0.902	141.8
50-54	195	255	881544	1015469	22.12	25.11		1.385	224.6
55-59	321	450	690245	981581	46.51	45.84		1.98	456.5
60-64	310	547	409084	724939	75.78	75.45		1.931	549.4
65-69	322	596	403032	536021	79.89	111.19		2.804	428.3
70-74	463	319	362684	261088	127.66	122.18		2.773	333.3
75-79	244	398	187376	288503	130.22	137.95		1.617	375.7
80-84	164	246	130769	155662	125.41	158.03		1.293	195.2
85+	40	57	55076	86664	72.63	65.77		0.227	62.9
Total	*n*_1_=2364	*n*_2_=3218	*N*_1_=15982370	*N*_2_=18157337	*P*_1_=14.79	*P*_2_=17.72	*P*_1_^C^ *=*14.79	*P*_2_^C^ *=*16.09	*E(n*_2_ *)*=2945
Growth									
Male*									
Total	*n*_1_=1131	*n*_2_=1555	*N*_1_=7698875	*N*_2_=8791298	*P*_1_=14.69	*P*_2_=17.69	*P*_1_^C^ *=*14.69	*P*_2_^C^ *=*15.93	E*(n*_2_*)*)=1443
Growth									
Female*									
Total	*n*_1_=1233	*n*_2_=1663	*N*_1_=8283495	*N*_2_=9366039	*P*_1_=14.89	*P*_2_=17.76	*P*_1_^C^ *=*14.89	*P*_2_^C^ *=*16.22	E*(n*_2_*)*=1506
Growth									

END, the expected number of diseases; *, The calculations were made in the same way as for the entire population.

**Table 4:** Influencing Components on the Number of Cases of CRC in Kazakhstan

Components of growth in the number of cases due to:	Both sexes			Male			Female		
	AN	% growth		AN	% growth		AN	% growth	
		to *(n*_2_*-n*_1_*)*	to *n*_1_		to *(n*_2_*-n*_1_*)*	to *n*_1_		to*(n*_2_*-n*_1_*)*	to *n*_1_
1. Growth PN	322	37.7	13.6	160	37.8	14.2	161	37.5	13.1
2. Changes ASP	227	26.6	9.6	132	31.2	11.7	99	23.1	8
3. Combined effect of changes in PN and ASP	31	3.6	1.3	19	4.4	1.7	13	3	1.1
	∑1-3=+67.8 ∑1-3=+24.5			∑1-3=+73.5 ∑1-3=+27.6			∑1-3=+63.5 ∑1-3=+22.2		
4. Change of RAI	210	24.6	8.9	95	22.5	8.4	110	25.7	9
5. Combined effect of changes of RAI and PN	29	3.3	1.2	14	3.2	1.2	14	3.4	1.2
6. Combined effect of changes of RAI and ASP	32	3.7	1.3	3	0.7	0.3	28	6.6	2.3
7. Combined effect of the changes RAI, PN and ASP	4	0.5	0.2	0	0.1	0.04	4	0.9	0.3
			
	∑1-3=+32.2 ∑1-3=+11.6			∑1-3=+26.5 ∑1-3=+9.9			∑1-3=+36.5 ∑1-3=+12.7		
Total ∑1-7	854	100	36.1	424	100	37.5	430	100	34.9

AN, absolute number; PN, population number; ASP, age structure of the population; RAI, risk of acquiring illness

**Table 5:** Component Analysis of CRC Incidence Growth with Respect to Regions of Kazakhstan, 2009-2018

Regions	Incidence,		Incidence growth, per 100,000				T, %	p	R^2^	AN	Change/Combined, %							Total
	per 100,000		general*	Including							Δ_P_	Δ_A_	Δ_PA_	Δ_R_	Δ_RP_	Δ_RA_	Δ_RAP_	
	2009	2018		Δ_A_	Δ_R_	Δ_RA_												
Almaty city	21.95	22.03	0.08	0.81	−0.99	0.25	−1.6	1	0.3475	98	98.6	11.3	3.6	−13.8	−4.4	3.5	1.1	100
North Kazakhstan	28.12	28.46	0.35	5.62	−2.72	−2.55	0.5	0.9	0.0649	−9	−121.7	373	−24.3	−180.4	11.8	−169.4	11	100
Pavlodar	29.37	30.2	0.84	3.79	−3.70	0.74	2.5	0.777	0.4236	10	36.9	281.6	4.8	−274.6	−4.7	55.1	0.9	100
Mangystau	6.84	7.88	1.04	1.1	−0.10	0.04	2.2	0.501	0.1759	19	63.9	27.9	10.3	−2.5	−0.9	1	0.4	100
Zhambyl	6.86	8.23	1.38	0.72	0.31	0.35	1.6	0.282	0.1656	22	30.1	33.5	3.2	14.2	1.3	16.2	1.5	100
South Kazakhstan	5.28	6.9	1.62	0.87	0.86	−0.12	4.2	0.024	0.5674	72	34.2	29.8	5.6	29.5	5.6	−4.0	−0.8	100
Kyzylorda	6.34	8.04	1.7	1.72	0.68	−0.70	1.4	0.232	0.0999	20	33.4	58.2	9.1	23.2	3.6	−23.9	−3.7	100
Astana city	18.01	19.99	1.98	2.27	−0.01	−0.28	1	0.364	0.0384	97	79	14.2	9.9	±0.0	±0.0	−1.8	−1.2	100
West Kazakhstan	17.88	20.71	2.83	2.06	0.97	−0.20	2.1	0.259	0.4035	27	32.2	45.7	3.7	21.5	1.7	−4.5	−0.4	100
Kazakhstan	14.79	17.72	2.93	1.42	1.31	0.2	2	0	0.7327	854	37.7	26.6	3.6	24.6	3.3	3.7	0.5	100
Almaty	7.76	11	3.24	0.6	2.43	0.21	3.5	0.001	0.2823	82	20.2	13.2	1.6	53.5	6.3	4.6	0.5	100
Atyrau	8.84	14.34	5.5	0.21	4.6	0.69	5.4	0.006	0.6244	44	22.4	2.4	0.5	53.2	11.7	8	1.8	100
Akmola	18.54	24.49	5.95	1.93	1.33	2.69	4.7	0.013	0.763	44	±0.0	32.5	±0.0	22.3	±0.0	45.1	±0.0	100
East Kazakhstan	22.55	28.76	6.21	3.09	3.07	0.04	2.7	0.001	0.6502	83	-3.6	52.1	−0.5	51.7	−0.5	0.8	±0.0	100
Karaganda	20.06	26.44	6.38	2.15	3.62	0.61	2.8	0.007	0.5941	96	8.2	30	0.9	50.6	1.5	8.5	0.2	100
Aktobe	10.17	17.02	6.85	1.83	4.23	0.79	4.1	0	0.6006	69	14.9	20	2.7	46.4	6.2	8.7	1.2	100
Kostanay	23.02	32.43	9.42	3.89	5.63	−0.11	3	0	0.5708	80	-3.1	43.1	−0.5	62.4	−0.8	−1.2	±0.0	100

*The table is built taking into account the sorting from A to Z of the general growth; T, average annual upward/downward rates; p, significance level; R^2^, the value of the approximation confidence; AN, absolute number; Δ_P_, population number; Δ_A_, the age structure of the population; Δ_PA_, population number and its age structure; Δ_R_, risk of acquiring illness; Δ_RP_, risk of acquiring illness and population number; Δ_RA_, risk of acquiring illness and age structure of the population; Δ_RAP_, risk of acquiring illness, population number and its age structure.

## Discussion

The results of this study showed that the incidence rate of CRC was as high as 16.9 cases per 100,000 in Kazakhstan, while the difference between men (16.7) and women (17.2) was not statistically significant in this regard. However, it was found that the ASRs was significantly higher in men (21.9) than women (15.9). Generally, the ASRs of the CRC was 18.1 cases per 100,00, revealing that Kazakhstan is moving into a zone of high incidence (Ferlay et al., 2020a). In terms of gender, the lowest rates in men was reported (per 100,000) in Mozambique (3.6) and Burkina Faso (3.6), and in women were reported in Bhutan (1.9) and Tajikistan (2.9). The highest rates of CRC in men were found in Slovakia (60.7) and Hungary (62.0), and in women were found in Denmark (35.6) and Norway (38.7) (Ferlay et al., 2020a).

The results on age-related incidence indicators in the country had high values in patients over 65 years of age. Similar results were found in French Polynesia (111.2 per 100,000), Jordan (114.8), Georgia (120.2), and United Arab Emirates (122.5). This pattern was repeated in men population and there were alike indicators in Saint Lucia (140.7), Qatar (141.2), Paraguay (147.9), and Costa Rica (147.5). Whereas in women, we saw changes similar to French Polynesia (96.4), Georgia (99.6), Venezuela (100.5), and Bahrain (101.2) (Ferlay et al., 2020A).

The results of the epidemiological study regarding CRC in Kazakhstan for 2004-2014 (Abdullayev et al., 2017) showed an increase in its incidence with stable trends, which was in line with our study. At the same time, there was an increase in the number of patients at stages I and II, which could be due to the influence of screening.

The incidence of CRC in Kazakhstan had increasing trend, which could be due to the impact of ongoing anti-cancer measures, including screening. Thus, according to Zhylkaidarova (2021), changes in morbidity can be estimated through the assessment of the impact of screening. Herewith, the regions that got a score of 0-2 points, according to our data, had a low degree of approximation reliability (i.e., changes in the trends were not significant). Our study revealed following results: Almaty city (0 points; R^2^=0.3475), North Kazakhstan (0 points; R^2^=0.0649), West Kazakhstan (1 point; R^2^=0.4035), Pavlodar (1 point; R^2^=0.4236), Kyzylorda (2 points; R^2^=0.0999), and Mangistau (2 points; R^2^=0.1759). Akmola (3 points; R^2^=0.7630), Kazakhstan (3 points; R^2^=0.7327), Aktobe (4 points; R^2^=0.6006), Karaganda (4 points; R^2^=0.5941), Kostanay (4 points; R^2^=0.5708), and South Kazakhstan (4 points; R^2^=0.5674) had a significant degree of approximation. In Atyrau (R^2^=0.6244) and East Kazakhstan (R^2^=0.6502), the degrees of approximation were significant. In Almaty (R^2^=0.2823), Zhambyl region (R^2^=0.1656), and Astana city (R^2^=0.0384), the degrees of accuracy of the approximation were not very pronounced, especially in the capital. These findings could be due to this fact that the authors did not study the growth rates of the dynamic series but compared the initial and final data of the studied period.

Having investigated the effectiveness of screening programs in the Almaty region for 2018-2020 (Zholmurzayeva et al., 2021), it was revealed that t the average response rate was 50% for the first step of screening and very low for the second step. At the same time, it should be noted that according to the data of medical and preventive institutions of the Almaty region, there was no excess load of the available endoscopic equipment. Consequently, the reasons for the insufficient response and screening were not related to the material problems of the healthcare system but to low social mobilization, which was similar to the findings of a study by Heisser (2021).

The results of our studies showed a unimodal increase in age indicators, which peaked at the age group of 75-79 years, and its incidence in this age group of men was almost twice as high. CRC incidence in the world in the age groups of 70 years and older was highest in Denmark (389.8 per 100,000 – both gender; 476.7 – male; 319.7 – female), Netherlands (390.9; 492.0; 306.1), and Norway (421.3; 473.3; 376.7) (Ferlay et al., 2020A).

With respect to gender-related differences, according to randomized study (Bull et al., 2020), higher BMI increases the risk of CRC among men, while higher waist-to-hip ratio considerably raises CRC risk among women. White et al., (2018) revealed that men were more prone to get CRC than women, though they see no gender differences in diagnosis for advanced stages of disease. Alongside this, the male population is more susceptible to a number of modifiable risk factors, such as smoking and alcohol intake (Rawla et al., 2019).

Declining trends of age indicators in women aged under 30, 30-34, 40-44, 50-59, 70-74 years old were notable. In other words, there was no increase in the diagnosis of this disease especially in screening target group (50-75 years). This phenomenon may be due to insufficient social mobilization, which needs health managers’ more attention. Meanwhile, a previous study revealed an association between reduction of morbidity and mortality and performing colonoscopy (Brown et al., 2021). They found that during the screening measures, there was a temporary increase (between 2002-2007) in CRC incidence, but in general (between 2000-2016), morbidity and mortality rates were declined. Authors consider such changes as expected ones because of detection of patients at early stage of CRC.

These findings can be attributed to the timely diagnosis and treatment of precancerous diseases. As in a previous study, a decrease in the incidence of CRC in the group subject to screening (50-74 year) was reported. The authors of aforementioned study attributed this phenomenon to the important role of screening in removal of adenomas and its notable effect on CRC incidence (Clark et al., 2020).

The formation and impact of the studied factors in regions were dissimilar. The analysis of data on the CRC incidence with respect to region showed that there were certain regions where the component of “risk of acquiring illness” had no effect. However, we found the presence of certain problems in the organization of anti-cancer measures, including screening.

In general, an increase in the incidence of CRC was noted in many regions, where this growth was due to the greater influence of the “risk of acquiring illness” component. It influenced the formation of the general trend in the country as a whole resulting from anti-cancer measures in CRC, including screening. Unfortunately, it was not possible to compare our component analysis results with the literature data due to the lack of research in this direction.

The results of component analysis indicated of the efficacy of anti-cancer measures, including CRC screening, in the country, while the influence of other exogenous and endogenous factors were not excluded.

## Author Contribution Statement

DM, AA, SS, YK, GN – Collection and preparation of data, primary processing of the material and their verification.

DM, SU, YZ, VO, NI – Statistical processing and analysis of the material, writing the text of the article (material and methods, results).

DM, SK, DT, ZB, GI, ZhB, SO – Writing the text of the article (introduction, discussion).

NI, AMJ, DM – Concept, design and control of the research, approval of the final version of the article. All authors approved the final version of the manuscript.
